# The Third Signal Cytokine IL-12 Rescues the Anti-Viral Function of Exhausted HBV-Specific CD8 T Cells

**DOI:** 10.1371/journal.ppat.1003208

**Published:** 2013-03-14

**Authors:** Anna Schurich, Laura J. Pallett, Marcin Lubowiecki, Harsimran D. Singh, Upkar S. Gill, Patrick T. Kennedy, Eleni Nastouli, Sudeep Tanwar, William Rosenberg, Mala K. Maini

**Affiliations:** 1 Division of Infection and Immunity, University College London, London, United Kingdom; 2 Centre for Hepatology, University College London, London, United Kingdom; 3 Centre for Digestive Disease, Barts and the London School for Medicine and Dentistry, London, United Kingdom; 4 Department of Clinical Microbiology and Virology, University College London Hospital, London, United Kingdom; Nationwide Children's Hospital, United States of America

## Abstract

Optimal immune activation of naïve CD8 T cells requires signal 1 mediated by the T cell receptor, signal 2 mediated by co-stimulation and signal 3 provided by pro-inflammatory cytokines. However, the potential for signal 3 cytokines to rescue anti-viral responses in functionally exhausted T cells has not been defined. We investigated the effect of using third signal cytokines IL-12 or IFN-α to rescue the exhausted CD8 T cell response characteristic of patients persistently infected with hepatitis B virus (HBV). We found that IL-12, but not IFN-α, potently augmented the capacity of HBV-specific CD8 T cells to produce effector cytokines upon stimulation by cognate antigen. Functional recovery mediated by IL-12 was accompanied by down-modulation of the hallmark inhibitory receptor PD-1 and an increase in the transcription factor T-bet. PD-1 down-regulation was observed in HBV but not CMV-specific T cells, in line with our finding that the highly functional CMV response was not further enhanced by IL-12.

IL-12 enhanced a number of characteristics of HBV-specific T cells important for viral control: cytotoxicity, polyfunctionality and multispecificity. Furthermore, IL-12 significantly decreased the pro-apoptotic molecule Bim, which is capable of mediating premature attrition of HBV-specific CD8 T cells. Combining IL-12 with blockade of the PD-1 pathway further increased CD8 functionality in the majority of patients. These data provide new insights into the distinct signalling requirements of exhausted T cells and the potential to recover responses optimised to control persistent viral infections.

## Author Summary

Persistent viral infections continue to cause major morbidity and mortality; chronic hepatitis B virus infection alone accounts for more than a million deaths annually. Such infections are characterised by a failure of viral control perpetuated by exhaustion of the T cell response. Here we show that the cytokine IL-12 can act as a potent “third signal” to rescue antiviral function in exhausted T cells. IL-12 has previously been shown to enhance naïve T cell responses but this is the first demonstration of its capacity to boost the disabled antiviral response in a persistent viral infection. IL-12 was able to down-regulate PD-1, a key inhibitory receptor driving T cell exhaustion, resulting in the recovery of hepatitis B virus-specific responses able to mediate multiple antiviral functions. Control responses in the same patients directed against the well-controlled cytomegalovirus did not require IL-12 to function efficiently. Our findings therefore elucidate a role for IL-12 in re-programming functionally exhausted T cells in persistent viral infections.

## Introduction

Successful T cell activation requires a T cell receptor (TCR)-mediated signal accompanied by a co-stimulatory signal through receptors such as CD28. In addition to these two signals, it is increasingly recognised that a third signal provided by the pro-inflammatory cytokines IL-12 and/or IFN-α can contribute to CD8 T cell activation [1]. Provision of a third signal during priming of naïve T cells prevents tolerance induction and cell death and is vitally important in shaping the memory response [1]. Although initially described to shape the lineage commitment of CD4 T cells and thereby indirectly influence CD8 T cells, it has become clear that IL-12 and IFN-αcan act directly on CD8 T cells, stimulating their activation [2], [3], [4]. In addition to their role in T cell priming, third signal cytokines are required for the reactivation of protective memory responses during secondary infections [5]. Whether a third signal cytokine can help to reactivate T cells exhibiting the characteristics of exhaustion in persistent viral infection has not been explored and is the focus of this study.

T cell exhaustion is characterised by a progressive, hierarchical loss of effector function, culminating in T cell deletion. Critical factors driving T cell exhaustion in the setting of persistent viral infection include high antigen load and an excess of co-inhibitory signals. Blocking co-inhibitory signals such as programmed death-1 (PD-1) and cytotoxic T lymphocyte antigen-4 (CTLA-4) and/or enhancing co-stimulation through receptors such as 4-1BB can restore some functional responses in persistent viral infection [6], [7], [8]. Blockade of inhibitory cytokines such as IL-10 and TGF-β has also been found to provide some reversal of T cell exhaustion [9], [10]. We postulated that the addition of a third signal cytokine would further augment the functional recovery of exhausted T cells.

We tested this postulate using T cells isolated from patients with chronic Hepatitis B virus infection (CHB). T cells in this setting are prone to Bcl-2 interacting mediator (Bim)-mediated apoptosis and are strikingly depleted in patients with chronic compared to resolved infection [11]. The remaining HBV-specific T cells exhibit the characteristics of exhaustion, with up-regulation of inhibitory molecules and down-regulation of effector function [12], [13]. Previous studies have demonstrated the potential to reconstitute functional HBV-specific T cells by blocking PD-1, CTLA-4 or both [12], [13]. However, there was incomplete rescue of HBV T cell specificities and not all patients responded to these strategies to enhance signal 2. Here we explored the potential for signal 3 cytokines to recover effector functions of CD8 T cells directed against chronic viral infections. We found that upon IL-12 stimulation, HBV-specific T cells down-regulated PD-1 expression and showed significantly increased antiviral potential upon recognition of cognate antigen. However, control T cell responses in the same patients, directed against CMV and not exhibiting features of exhaustion, did not show changes in PD-1 expression or increased anti-viral responses above those triggered by cognate antigen alone. Our data therefore suggest that IL-12 is particularly beneficial for enhancing TCR-mediated signalling in T cell responses that are exhausted. Furthermore, IL-12 can significantly augment the effects of co-inhibitory blockade.

## Results

### Differential effect of third signal cytokines on HBV-specific CD8 T cells

**Figure 1:**
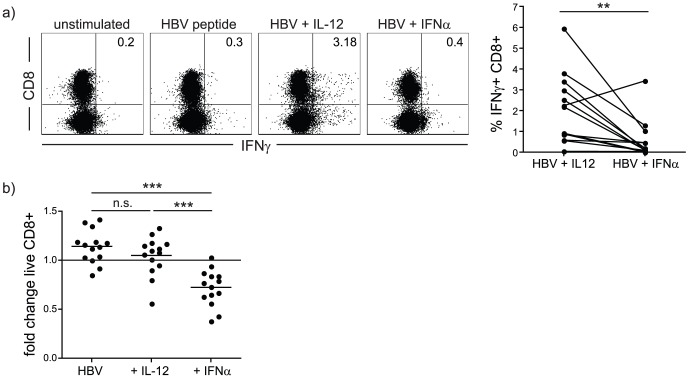
IL-12 but not IFN-α increases IFN-γ production by HBV-specific CD8 T cells. a) PBMC from CHB patients were stimulated with HBV derived peptides and cultured for 10 days in the presence or absence of IL-12 or IFN-α. Cells were then restimulated with HBV-derived peptides overnight and IFN-γ production from CD8+ T cells was quantified by intracellular FACS. Representative FACS plots and summary data are shown. b) Cumulative data showing the fold change in cell yield compared to control unstimulated PBMCs cultured in the absence of HBV peptide. Numbers of CD8 T cells were measured by using a live/dead staining kit to exclude dead cells after 10 days culture.

CD8 T cells able to produce IFN-γ upon recognition of HBV epitopes are markedly reduced in patients with persistent infection [14], [15]. We investigated the capacity of the third signal cytokines IL-12 and IFN-α to rescue functional HBV-specific CD8 T cell responses. PBMC from chronic hepatitis B (CHB) patients were stimulated with HBV peptides (a panel representing well-defined HLA-A2-restricted epitopes or overlapping peptides (OLP) spanning the entire core protein), and cultured in IL-2-enriched medium supplemented with either IL-12 or IFN-α. The addition of IFN-α did not recover any IFN-γ-producing HBV-specific CD8 T cells (Figure 1a). By contrast, CD8 T cells cultured in the presence of IL-12 showed a marked increase in IFN-γ production above those stimulated with peptide with or without IFN-α (Figure 1a). Global CD8 T cell yield in the presence of IFN-α was also significantly reduced, as assessed by numbers of live CD8 T cells detected at the end of culture after dead cell exclusion, whereas their numbers were relatively preserved with IL-12 (Figure 1b). These results suggested that IL-12 is a more potent third signal than IFN-α in stimulating CD8 effector function and preserving T cell numbers in the setting of CHB.

**Figure 2:**
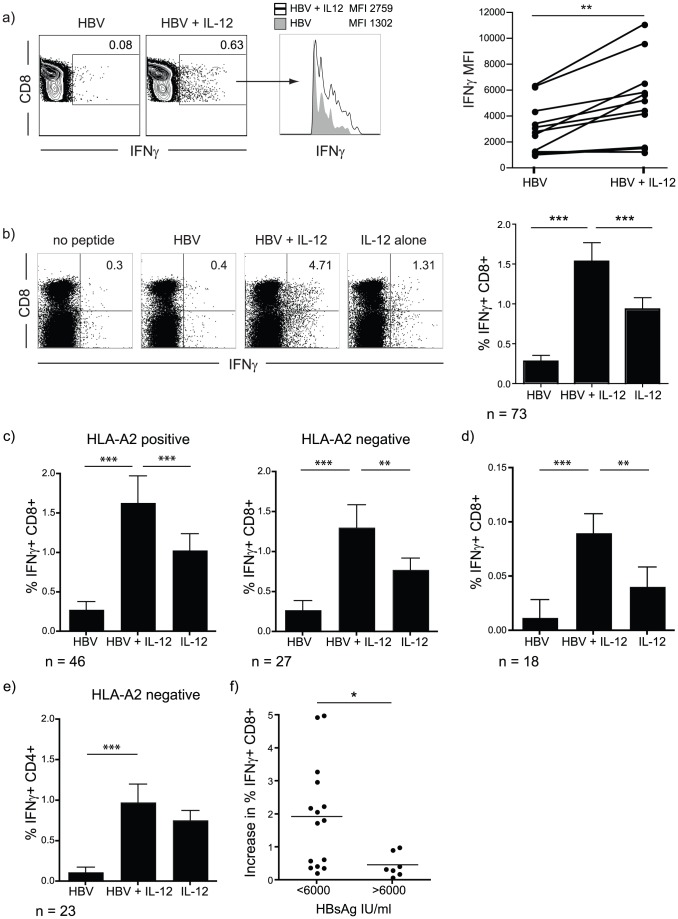
IL-12 in combination with T cell receptor stimulation increases IFN-γ production irrespective of HLA-type but dependent on HBsAg levels. a) The effect of IL-12 on IFN-γ production on a per cell basis was measured by changes in IFN-γ MFI after restimulation of PBMC cultured for 10 days with HBV peptides in the presence or absence of IL-12. Representative FACS plot gated on CD8 T cells, IFN-γ-overlay histogram and summary data. b) Representative FACS plot and summary data of CD8 T cell IFN-γ production after 10 days culture with HBV peptides, HBV peptides with IL-12, or IL-12 in the absence of peptide, background IFN-γ produced in corresponding unstimulated control samples has been subtracted. c) Data from Figure 2b were split into responses produced by HLA-A2 positive (stimulated with HLA-A2 restricted immunodominant peptides) and HLA-A2 negative (stimulated with core OLP) samples. d) PBMC were stimulated over night with HBV-derived peptides in the presence or absence of IL-12, in the presence of Brefeldin A. IFN-γ production was assessed by intracellular staining. e) PBMC from HLA-A2 negative patients were stimulated in the presence or absence of core OLP and/or IL-12 and cultured for 10 days, before peptide restimulation. T cells were gated on the CD3 positive CD8 negative fraction (denoted as CD4+) and IFN-γ production assessed by intracellular staining. f) Patients were stratified according to serum HBsAg levels (available for n = 22, divided according to a threshold of 6000 IU/ml) and the increase in IFN-γ response frequency upon HBV+ IL-12 stimulation above stimulation by HBV-peptide alone is shown (%IFN-γ (HBV+IL-12) - % IFN-γ (HBV)).

We next investigated whether exogenous IL-12 was able to increase not only the frequency of IFN-γ+ T cells but also the amount of IFN-γ produced on a per cell basis. We observed that IL-12 was able to consistently increase the amount of IFN-γ produced per cell, indicated by an increase in IFN-γ mean fluorescence intensity (MFI) in CD8 T cells responding to HBV peptide stimulation (Figure 2a).

Since IL-12 in combination with IL-18 has been reported to trigger some bystander production of IFN-γ by murine CD8 T cells [2], [4], we extended our examination of the effect of IL-12 to a larger cohort of 73 patients with CHB, comparing the addition of IL-12 with or without HBV peptides (Figure 2b). The combination of HBV peptides with IL-12 (providing signal 1 and 3) stimulated significantly more IFN-γ+ cells than either HBV peptide or IL-12 alone (Figure 2b). This finding was true whether optimal HLA-A2-restricted or overlapping peptides spanning HBV core (to stimulate T cells derived from HLA-A2 negative patients) were used (Figure 2c). To validate that the boosting effect of IL-12 was not only effective after short-term culture of CD8 T cells, PBMC were stimulated with HBV-derived peptides in the presence or absence of IL-12 overnight. Responses to HBV-derived peptides alone were, as expected in CHB, very weak, but even at this early time point IFN-γ responses were enhanced by IL-12 (Figure 2d). This finding suggested that virus-specific T cells present in peripheral blood could recover some functionality. Of note, IL-12 did induce an increase in IFN-γ+ CD8 T cells in overnight and short-term cultures in the absence of peptide, suggesting it may also activate responses in a non-antigen-dependent manner (Figure 2b, c and d). CD4 T cells also showed increased IFN-γ production when stimulated with IL-12. This augmentation was, however, non-specific since stimulation with overlapping peptides spanning HBV core had no influence on the magnitude of the response (Figure 2e).

To probe the potential of IL-12 to boost functional HBV-specific CD8 T cell responses within the liver, we isolated intrahepatic lymphocytes (IHL) from six patients with CHB. Four out of six patients showed an increased HBV response upon IL-12 treatment of liver infiltrating lymphocytes after overnight stimulation; in all cases the effect of IL-12 was more pronounced on IHL than on PBMC from the same patients (Figure S1).

In order to investigate the utility of IL-12 to rescue HBV-specific CD8 T cells in an immunotherapeutic setting, we compared its efficacy *in vitro* using samples from patients undergoing antiviral therapy (Table SI). PBMC derived from four patients with CHB were tested for responsiveness to IL-12 before and during potent antiviral therapy. In all four cases CD8 T cells remained responsive to IL-12 to a greater or lesser extent during treatment (Figure S2). In one case anti-HBV responses could only be detected on treatment and were greatly augmented by IL-12.

The extent of CD8 T cell functional augmentation achieved with IL-12 stimulation did not correlate with viral load, alanine transaminase (ALT) or hepatitis B eAg (HBeAg) status in our large patient cohort (data not shown). However recovery of responses upon IL-12 stimulation did correlate with circulating levels of hepatitis B surface antigen (HBsAg); patients with an HBsAg titre less than 6000 IU/ml showed variable IL-12 responsiveness, with the potential for large increases in functional HBV-specific CD8 T cells. By contrast, none of the patients in whom HBsAg was greater than 6000 IU/ml showed a substantial augmentation upon IL-12 stimulation (Figure 2f).

### IL-12 boosts antigen responsiveness by HBV-specific but not CMV-specific CD8 T cells

**Figure 3:**
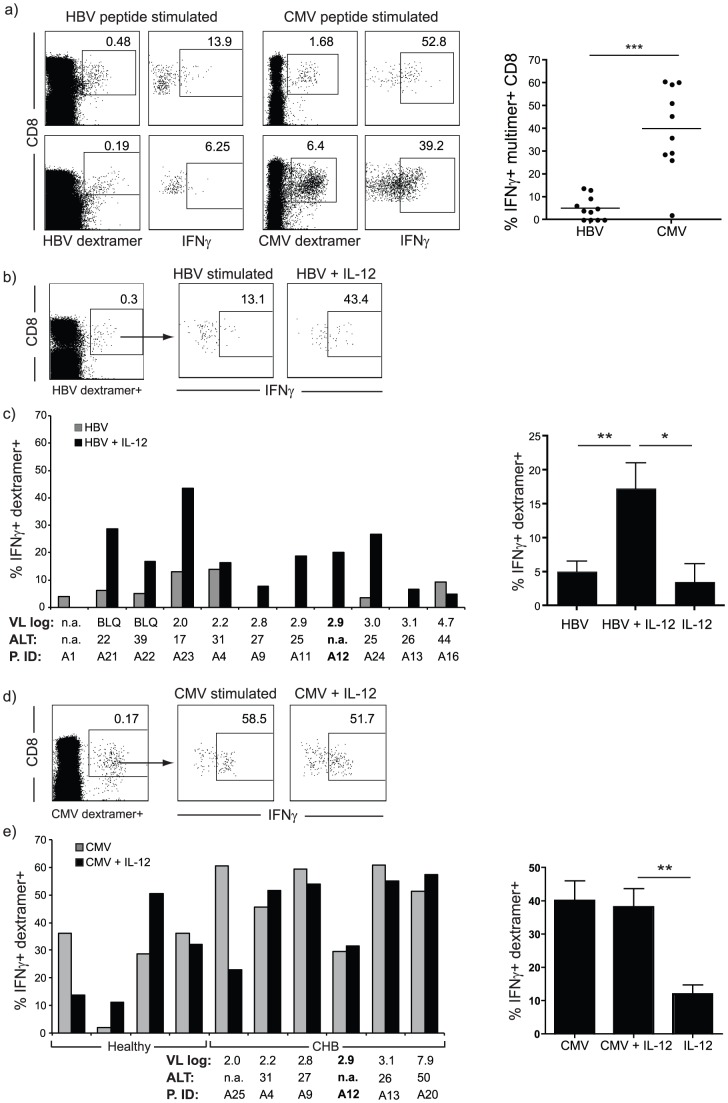
Influence of IL-12 on IFN-γ production by HBV and CMV-specific CD8 T cells. a) HBV- and CMV-specific CD8 T cells were detected by HLA-A2/peptide dextramer staining and IFN-γ production by the gated dextramer-positive cells was determined by intracellular FACS. FACS plots gated on live CD19^−^ CD3^+^ CD8^+^ T cells for HBV and CMV responses from 2 representative patients and summary data of the proportion of dextramer-staining CD8 able to produce IFN-γ are shown. b) Influence of IL-12 on IFN-γ production by HBV dextramer+ T cells stimulated with corresponding peptides. Representative FACS plot showing IFN-γ production by gated dextramer+ T cells in the presence or absence of IL-12 and c) summary data for each patient according to HBV viral load (log_10_IU/ml). ALT (IU/L) and patient identifier (P. ID) are shown; samples from patients with HBeAg+ CHB are in bold (left) and combined for whole cohort (right); abbreviations: n.a. information not available, BLQ below limit of quantification. d) IFN-γ production by T cells gated on the CMV NLV-dextramer+ fraction in the presence or absence of IL-12, representative FACS stain and e) frequency of IFN-γ+ CMV NLVP-dextramer+ T cells from all individuals tested are shown (derived from healthy controls and CHB patients as denoted).

To further investigate the antigen specificity of IFN-γ boosting by IL-12, we identified virus-specific T cells with MHC-peptide multimers and assessed the capacity of these populations to produce IFN-γ upon peptide stimulation. PBMC from patients with CHB were cultured for ten days in the presence of peptides representing well-described HLA-A2-restricted HBV epitopes or for comparison, an immunodominant HLA-A2-restricted CMV epitope, and stained with HLA-A2-peptide dextramers of matched specificity. To prevent false positive events from contaminating the low frequency of genuine HBV/multimer-staining CD8 T cells, we excluded dead cells and B cells using a fixable live/dead dye and CD19 respectively (Figure S3). The small populations remaining after this stringent gating strategy able to bind the combined panel of HLA-A2/HBV peptide dextramers were barely able to produce any IFN-γ (Figure 3a). This was in contrast to CD8 T cells specific for CMV, which showed robust IFN-γ production when stimulated with their cognate peptide (Figure 3a).

These experiments were repeated with or without the addition of IL-12 at the start of the ten day culture. IL-12 did not have any consistent effect on the number of dextramer positive cells expanding in culture (Figure S4). However, IL-12 was able to recover IFN-γ production by HLA-A2/HBV peptide dextramer-binding CD8 T cells in the majority of patients tested (regardless of viral load, ALT or eAg status), in some cases restoring it to proportions analogous to control CMV responses (Figure 3b,c). In contrast to the effect on global T cells, IL-12 alone in the absence of peptide could only marginally stimulate HBV multimer-binding CD8 T cells (Figure 3c). These results indicate that HBV-specific, exhausted T cells benefit from receiving a signal 3 (IL-12) but also need stimulation via the TCR (signal 1) to recover functionality. The stimulatory effect of IL-12 could be seen as early as the first day of culture. To assess whether IL-12 could induce IFN-γ production by HBV-specific T cells directed against the epitopes derived from the different HBV proteins, PBMC from HLA-A2 positive patients were stimulated with either core, envelope or polymerase derived peptides in the presence or absence of IL-12. Staining with the matched HLA-A2/HBV peptide dextramers showed that IL-12 had the capacity to increase IFN-γ production by T cells directed against core, envelope and polymerase proteins (Figure S5).

To investigate whether boosting of responses by IL-12 was a feature of all HBV-specific responses regardless of the disease setting, we stimulated CD8 T cells from patients who had previously resolved HBV infection with HBV-derived peptides in the presence or absence of IL-12. The vigorous IFN-γ response triggered by peptide recognition alone could not be significantly enhanced in the CD8 T cells from resolved patients, suggesting that the requirement for additional stimulation by IL-12 is specific to the exhausted CD8 T cell responses in chronic infection (Figure S6). To further examine whether the dependence of HBV-specific T cells on signal 3 was a feature of their exhausted phenotype or was shared by T cells of other specificities circulating in patients with CHB, we assessed the effect of IL-12 on CMV-reactive T cells. IL-12 had no influence on IFN-γ production by CMV dextramer-binding T cells, resulting in no increase above that seen with peptide stimulation alone (Figure 3d). The lack of IL-12-induced augmentation of IFN-γ production by CMV-specific CD8 T cells was seen regardless of whether the donors were healthy or had CHB (Figure 3e). Of note, IL-12 alone could trigger IFN-γ production by approximately 10% of CMV-specific T cells in the absence of cognate peptide (Figure 3e, right panel), suggesting that these prevalent responses may contribute to the bystander effect we had observed with IL-12.

### Pleiotropic effect of IL-12 in restoring functional HBV-specific CD8 T cells

**Figure 4:**
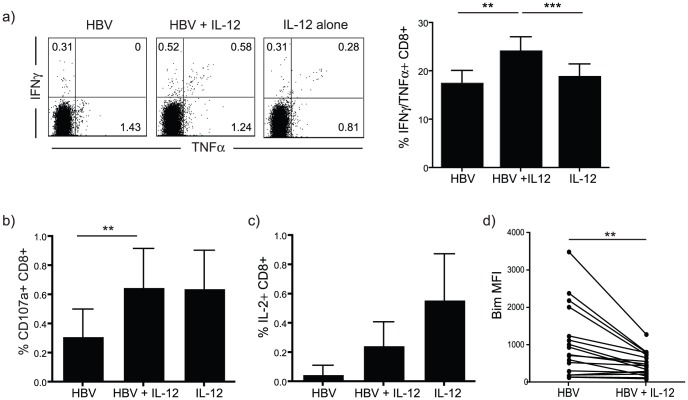
IL-12 promotes polyfunctional responses by HBV-specific T cells. a) Representative FACS plot showing IFN-γ and TNF-α production after restimulation of CD8 T cells with HBV-derived peptides. The summary data show the percentage of IFN-γ/TNF-α double positive CD8 T cells out of all those producing either cytokine. b) Frequency of CD107a and c) IL-2 positive CD8 T cells upon restimulation with HBV-derived peptide, cells were cultured in the presence or absence of IL-12. d) Expression levels of the pro-apoptotic molecule Bim in HBV-specific **(**IFN-γ+) CD8 T cells after culture in the presence or absence of IL-12; mean fluorescence intensity (MFI) is shown. All experiments were analysed after 10 day culture.

CD8 T cells that are able to simultaneously exert a number of different effector functions have been shown to be important in overcoming persistent viral infections [16], [17]. Very few of the HBV-specific CD8 T cells detectable using IFN-γ and TNF-α as a readout after peptide stimulation were able to produce both cytokines (Figure 4a). The addition of IL-12 was able to significantly boost both the frequency (data not shown) and the proportion of IFN-γ/TNF-α double-positive CD8 T cell responses (Figure 4a). Although IFN-γ and TNF-α can potently control HBV replication in a non-cytolytic manner, CD8 cytotoxicity is likely to be required for the final clearance of replicative intermediates [18]. T cells stimulated with IL-12 also showed an increase in their capacity for cytotoxic degranulation, as evidenced by an upregulation in surface expression of CD107a, which was independent of peptide stimulation (Figure 4b). In accordance with previously published work [19] and in line with the hierarchical loss of effector function characteristic of exhaustion [20], IL-2 production by HBV-specific T cells was rarely detected and was not recovered upon stimulation with IL-12 (Figure 4c). IL-12 was, however, able to induce the survival of a population of HBV-specific CD8 T cells exhibiting reduced expression of the pro-apoptotic molecule Bim (Figure 4d), previously implicated in their premature attrition [11].

### IL-12 stimulation leads to down-regulation of the co-inhibitory molecule PD-1

**Figure 5:**
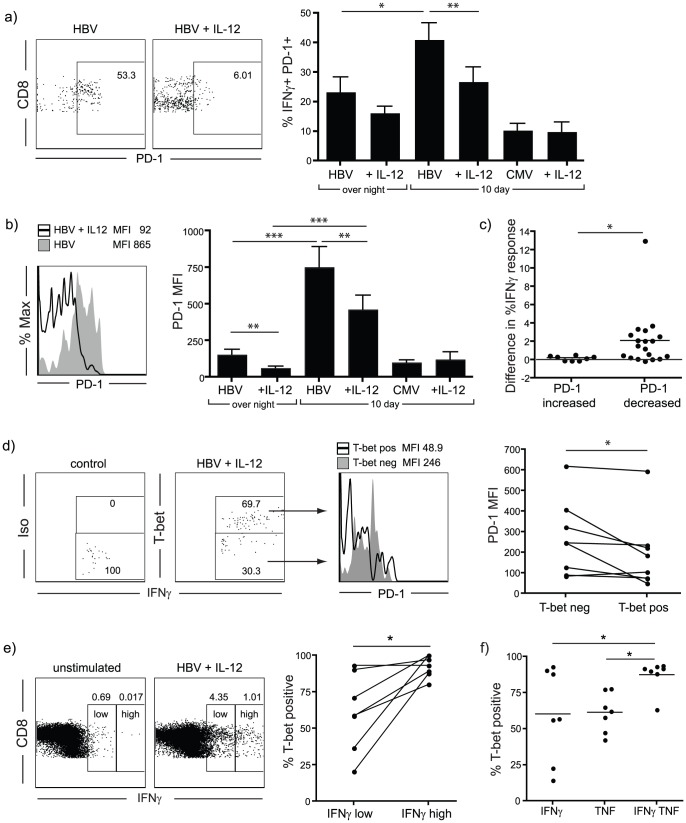
Influence of IL-12 on PD-1 expression in HBV- and CMV-specific T cells. a) Frequency of PD-1+ HBV- or CMV-specific T cells (defined as CD8+ IFN-γ+) and (b) PD-1 expression levels as measured by MFI after short term culture in the presence or absence of IL-12, example FACS plots and summary data are gated on CD8+ IFN-γ+ T cells (n = 16 for overnight HBV, n = 27 for HBV- and n = 9 for CMV-specific 10 days). c) PD-1 MFI was compared on IFN-γ+ CD8+ T cells after stimulation with HBV-derived peptide in the presence or absence of IL-12. Samples were then divided according to whether PD-1 MFI decreased or not upon IL-12 stimulation. The graph shows the change in the frequency of IFN-γ producing cells upon IL-12 addition compared to peptide stimulation alone. d) PBMC were cultured with HBV derived peptides in the presence of IL-12; IFN-γ positive cells were divided into T-bet positive and negative cells according to an isotype staining control performed on IL-12 treated cells. PD-1 expression (mean fluorescence intensity) was compared in both groups. e) IFN-γ positive T cells were divided into IFN-γ low and IFN-γ high positive cells, defined by background IFN-γ production by CD8+ T cells in unstimulated samples. T-bet expression in IFN-γ low and high producing T cells was compared in samples tested after stimulation with HBV peptide and IL-12. f) Frequency of T-bet positivity in T cells producing either IFN-γ or TNF-α alone or co-producing both cytokines.

The co-inhibitory molecule PD-1 is a marker of exhaustion that is highly expressed on virus-specific T cells in persistent viral infections, including CHB [14], [21] (Figure 5a,b). We found that the upregulation in frequency and intensity of PD-1 expressing HBV-specific CD8 T cells seen upon culture was significantly reduced in the presence of IL-12 (Figure 5a,b). In contrast, CMV-specific T cells, which are not considered to be functionally exhausted [22], expressed considerably lower levels of PD-1 compared to HBV-specific T cells in the same CHB patients. CMV-specific T cells did not show a decrease in PD-1 expression upon IL-12 stimulation (Figure 5 a,b).

Strong increases in IFN-γ production upon stimulation with HBV-derived peptide in combination with IL-12 could only be observed in those samples with decreased expression of PD-1 (compared to the group treated with peptide alone, Figure 5c and individual responses, Figure S7).

In a mouse model of chronic LCMV infection, the transcription factor T-bet has recently been shown to counteract CD8 T cell exhaustion by down-regulating PD-1 and sustaining effector cytokine production in virus-specific cells [23]. In accordance with this, we found reduced expression of PD-1 on HBV-specific CD8 T cells with high levels of T-bet (Figure 5d). As T-bet transactivates IFN-γ gene expression we sought to evaluate whether it played a role in the increased functionality of IL-12 treated cells. When the IFN-γ+ CD8 T cell population was subdivided into high and low IFN-γ producers, those responding to IL-12 with the most efficient IFN-γ production were found to express uniformly high levels of T-bet (Figure 5e). Furthermore, all the IFN-γ/TNF-α double-producing CD8 T cells obtained after culture in the presence of HBV peptides and IL-12 were T-bet positive, whilst the single producers showed lower frequencies of T bet expression (Figure 5f).

### IL-12 enhances the capacity of co-inhibitory blockade to rescue exhausted T cells

**Figure 6:**
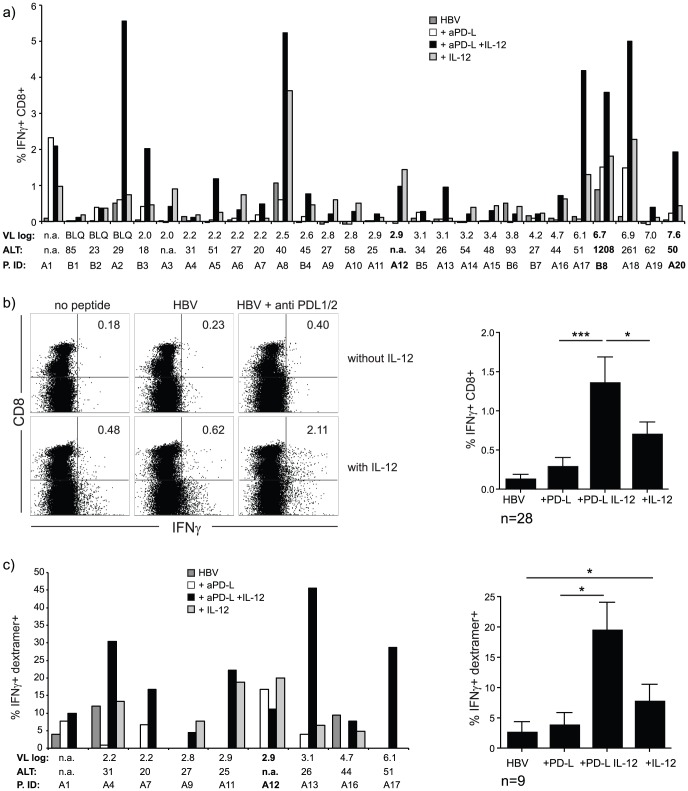
Blocking PD-1 signalling in combination with IL-12 enhances virus-specific responses. a) % IFN-γ+CD8+ upon peptide stimulation +/−PD-L blockade and IL-12 stimulation for all patients tested, presented for each patient in order of increasing viral load (VL) (log_10_IU/ml), ALT indicated in IU/L and eAg+ samples are shown in bold. Patient identifiers start with A for HLA-A2+ and with B for HLA-A2− donors. b) Representative FACS plots showing IFN-γ production upon restimulation with HBV derived peptides after blockade of PD-L1 and PD-L2 by a specific antibody, IL-12 stimulation or the combination and graph showing summary data for all patients. c) Functional recovery of HLA-A2/HBV dextramer+ T cells upon PD-L1/2 blockade, IL-12 stimulation or the combination. (VL (log_10_IU/ml), ALT (IU/L), patient ID (P. ID), n.a. = not available, BLQ = below limit of quantification. All data were acquired after 10 days culture and peptide restimulation overnight.

Although IL-12 could significantly decrease PD-1 expression on HBV-specific T cells, levels were still not as low as on control CMV-specific T cell responses (Figure 5a,b). We therefore postulated that blocking residual PD-1 signalling whilst stimulating with IL-12 would synergistically recover exhausted T cells. Blockade of inhibitory signalling via PD-L1/2 without IL-12 increased T cell IFN-γ production in 11 of the 28 patients tested (Figure 6a,b), in line with previous studies showing that not all patients show a functional recovery with this strategy [13]. However, when PD-L1/2 blockade was combined with IL-12 stimulation, IFN-γ production was enhanced in all but one patient, irrespective of ALT, eAg status or viral load (including all five patients with viral load above 1 million IU/ml, Figure 6a,b). Furthermore, the combination had a beneficial effect over IL-12 alone in 14 out of 28 patients (Figure 6a,b). To validate that this additional effect was not due to bystander activation, but to the recovery of functional HBV-specific T cells, we assessed IFN-γ production by HBV dextramer+ CD8 T cells after stimulation with IL-12 and PD-L1/2 blockade. The results confirmed that HBV-specific T cell function was significantly enhanced by the addition of IL-12 to PD-L1 blockade, with six out of nine patients showing optimal recovery when treated with the combination of PD-L1/2 blockade and IL-12 (Figure 6c).

## Discussion

Multiple mechanisms combine to drive T cell exhaustion and limit effective antiviral responses in the setting of chronic viral infections. The combination of a persistently high viral load and the tolerogenic liver environment promotes many such mechanisms, with intrahepatic priming imposing a Bim^high^ pro-apoptotic phenotype on T cells, whilst the excess of co-inhibitory signals from liver resident cells impairs their effector function [24]. We found that the signal 3 cytokine IL-12 can overcome these defects to recover polyfunctional, multispecific CD8 T cell responses with reduced levels of Bim and PD-1. Exhausted T cell responses directed against HBV in patients with persistent infection benefitted from the addition of IL-12 to signal 1 whereas they did not respond to IL-12 alone. Conversely, T cells that had not been driven to a state of exhaustion (HBV-specific T cells in patients with resolved infection or CMV responses in patients with persistent HBV infection) were more responsive to the bystander effects of IL-12 but did not require it to enhance signal 1. Our data therefore provide new insights into the distinct signalling requirements of exhausted T cells and reveal a strategy to recover T cell responses with the potential to control persistent viral infections.

Stimulation with another signal 3 cytokine, IFN-α, did not recover any IFN-γ-producing HBV-specific CD8 T cells *in vitro*, in line with the failure of therapeutic IFN-α to recover functional responses against HBV *in vivo* [25]. By contrast, IL-12 has the potential to reduce viraemia through the induction of IFN-γ-producing T cells in the HBV transgenic mouse model [26], in woodchuck hepatitis virus infection [27] and in patients with CHB [28], [29]. Exploring the mechanism of action of IL-12 on exhausted HBV-specific CD8 T cells, we found that it potently increased IFN-γ and TNF-α production and enhanced their capacity to produce both cytokines simultaneously. IL-12 also boosted CD8 cytotoxicity but could not consistently increase IL-2 or T cell expansion. This is consistent with the observation that exposure of naïve human T cells to IL-12 *in vitro* preferentially leads to formation of CD8 effector memory cells that efficiently produce effector cytokines but are poor at proliferative expansion, whereas IFN-α induces CD8 central memory cells [30].

The development of end stage effectors during LCMV infection is instructed by pro-inflammatory signals from IL-12, which acts via induction of the transcription factor T-bet [31]. In CD8 T cells, T-bet is expressed in response to TCR-signalling causing a positive feedback loop, with T-bet increasing IFN-γ, cytotoxicity and IL-12 receptor expression [32]. In line with this, we found the highest levels of T-bet in those CD8 T cells recovering maximal cytokine productivity following IL-12 stimulation. In support of a role for T-bet in the function of exhausted T cells, it has recently been implicated in supporting effector cytokine production and down-regulation of the co-inhibitory receptor PD-1 in chronic LCMV infection in mice [23]. We found that IL-12 stimulation of HBV-specific CD8 T cells resulted in the expansion of a population with significantly lower levels of PD-1, correlating with their recovery of function. However, PD-1 expression by IL-12-stimulated HBV-specific CD8 T cells remained elevated compared to levels on CMV-specific CD8 T cells and the addition of PD-L1 blockade was able to further optimise the rescue of antiviral responses. Prolonged exposure to IL-12 has recently been suggested to upregulate the Tim-3 pathway in non-Hodgkin lymphoma [33], lending further support to the strategy of combining IL-12 with co-inhibitory pathway blockade for maximal effect.

IL-12 has previously been found to activate sustained IFN-γ production by murine memory T cells without the need for antigen recognition [2], [4] analogous to the responses we saw by global CD8 T cells and CMV-specific memory responses upon stimulation with IL-12 in the absence of peptide. Although T cells specific for the well-described HBV epitopes we focused on could not be triggered to increase IFN-γ by IL-12 alone, we cannot exclude the possibility that CD8 T cells specific for other subdominant HBV epitopes, which may constitute less exhausted responses, could contribute to the bystander production. Recent work suggests that IFN-γ production by bystander CD8 T cells may contribute to viral clearance in acute infections [34]; boosting such bystander responses in CHB could potentially aid viral control. Additionally the Th1 type CD4 response stimulated by IL-12 may provide CD4 help in a non-antigen specific manner to support HBV-specific CD8 T cells. In contrast to HBV-specific T cells, IL-12 could not increase IFN-γ production by CMV-specific T cells once they had recognised their cognate antigen. This suggests there are different requirements in the activation of exhausted versus senescent T cells, respectively, strengthening the notion that these two processes are independently regulated [22].

In summary, our data suggest that exhausted T cells may no longer be amenable to activation by pro-inflammatory cytokines alone but benefit from stimulation with IL-12 together with cognate peptide. IL-12-treated T cells exhibited lower levels of Bim and PD-1, both of which play a critical role in curtailing responses that have been primed in the liver [11], [24], [35], [36]. IL-12 was able to increase cytolytic and non-cytolytic responses and enhance their multispecificity, even recovering responses directed against epitopes from the HBV envelope antigen that cannot be rescued by blockade of the PD-1 pathway [14]. Whereas blockade of the PD-1 [14] or CTLA-4 [13] co-inhibitory pathways rescued HBV-specific CD8 T cells in a proportion of patients, the addition of IL-12 enhanced the functionality of responses in significantly more patients, even those with extremely high viral loads. Patients who had HBV viraemia well-suppressed on antivirals were still responsive to IL-12 stimulation *in vitro*, suggesting that this may form a rational addition to a therapeutic vaccine in this setting. Such an approach has been used in macaque SIV infection, where the addition of IL-12 to an SIV DNA vaccine boosted effector memory CD8 T cells able to co-produce IFN-γ/TNF-α [37]. Future studies will reveal whether our strategy of combining antigenic stimulation, co-inhibitory blockade and IL-12 can optimise the rescue of exhausted T cells in other persistent viral infections.

## Materials and Methods

### Patients and controls

Ethics statement: This study was approved by the local ethical board of the Royal Free Hospital and Camden Primary Care and written informed consent was obtained from all participants. A total of 98 patients with CHB and 4 healthy volunteers participated in the study. All participants were HCV and HIV sero-negative. All patients were treatment naïve at the time of the study unless otherwise specified. CHB patients were stratified by eAg status, HBV DNA levels (determined by real-time PCR) and HBsAg titre (quantified with the Architect assay, Abbot Diagnostic). HLA-A2 status was determined by specific antibody (AbD Serotec).

### Over night and short-term cell culture and stimulation

PBMC were isolated by Ficoll-Hypaque density gradient centrifugation and either analysed directly or cryopreserved. To examine the effect of IL-12 and IFN-α on virus-specific T cells, PBMCs from patients or CMV+ healthy controls were cultured for 10 days before analysis. Briefly, At the start of the culture PBMCs from HLA A2+ donors were stimulated with 1 µM HBV-derived HLA-A2 restricted peptides (core FLPSDFFPSV, envelope FLLTRILTI, WLSLLVPFV, LLVPFVQWFV, GLSPTVWLSV, polymerase GLSRYVARL, KLHLYSHPI) (Proimmune) or if derived from HLA-A2 negative donors with 1 µM OLP spanning the whole HBV core protein, sequence correlating to HBV genotype D (AYW) (JPT Peptide Technologies). OLP spanning only HBV core were used to minimise inter-genotypic sequence variation. Control responses to CMV from HLA-A2+ individuals were measured using 1 µM NLVPMVATV (Proimmune). At the same time point rhIL-12 (Miltenyi Biotech) was added at 10 ng/ml, rhIFN-α (PBL Biomedical Laboratories) at 1000 IU/ml and function blocking anti-PD-L1, anti-PD-L2 and control IgG (eBiosciences) were used at 5 µg/ml. All cultures were supplemented with 20 U/ml rhIL-2 (Miltenyi Biotech) at day 0 and 4. PBMCs were restimulated on day 9 by re-adding peptide at the original concentration and culturing over night in the presence of 1 µg/ml Brefeldin A (Sigma-Aldrich). Virus-specific responses were identified by IFN-γ production. For detection of CD107a 1 µg/ml Monensin (Sigma-Aldrich) was also added during restimulation. To assess ex vivo IFN-γ+ responses, PBMC were stimulated overnight with 5 µM of HBV peptides either representing HLA-A2 restricted epitopes or spanning HBV core (as described above). IL-12 was used at 10 ng/ml. Cultures were not supplemented with IL-2. Brefeldin A was added 1 h after addition of peptide and IL-12.

### Intrahepatic lymphocyte isolation and functional assay

Liver sections from biopsies were homogenised and filtered. PBMC and liver cell suspensions from the same patient were stimulated over night with core OLP in the presence or absence of IL-12, in the presence of Brefeldin A. Virus-specific responses were identified by IFN-γ production as described for PBMC above.

### Flow cytometric analysis

9 or 10 colour flow cytometry was used for all experiments. PBMCs were stained for surface markers CD3, CD8 (eBioscience) and PD-1 (Biolegend). Dead cells were always excluded using live/dead fixable dye staining kit (Invitrogen). Cells were then fixed and permeabilized and intracellular molecules were detected using anti IFN-γ, TNF-α, CD107a (BD Biosciences), IL-2, Tbet (eBioscience) and Bim (Alexis). All samples were acquired on a BD LSRII or BD Fortessa. All analysis was performed using Flowjo (Tree Star).

### Staining for multimer+ T cells and intracellular cytokine production

HBV and CMV-specific HLA-A2 dextramers (Immudex) were titrated to determine optimal staining concentrations. For detection of HBV-specific T cells we used core FLPSDFFPSV, envelope FLLTRILTI, WLSLLVPFV, GLSPTVWLSV, polymerase GLSRYVARL, KLHLYSHPI. CMV-specific dextramers were loaded with NLVPMVATV; as a control for unspecific staining, dextramer loaded with irrelevant peptide was used (Figure S3b). Briefly, cells were stained with dextramers, washed and stimulated with peptide for 1 h before the addition of Brefeldin A. After 5 h incubation cells were stained with the surface marker antibodies, live/dead staining and anti-CD19 (BD Biosciences) to exclude dead cells and non-specific binding, respectively. Intracellular cytokines were detected as described.

### Statistical analysis

Statistical analyses were performed using the non-parametric Mann-Whitney or Wilcoxon matched pairs test as appropriate and significant differences marked on figures (* = p<0.05; ** = p<0.005; *** = p<0.0005).

## Supporting Information

Figure S1 **IL-12 enhances IFN-γ production in IHL overnight.** PBMC and IHL derived from the same patient were stimulated with core OLP over night in presence or absence of IL-12. IFN-γ production was assessed by intracellular cytokine staining. a) Example FACS plot comparing IFN-γ production by CD8 T cells derived from PBMC and IHL and b) summary data of all patients tested. VL (IU/ml) is shown as log_10_, all patients were eAg negative.
(EPS)

Figure S2 **Effect of IL-12 on IFN-γ production by HBV-stimulated CD8 is maintained on antiviral treatment.** PBMC from patients before and after starting antiviral treatment were stimulated with HBV derived peptides with or without IL-12 for 10 days, before restimulation overnight. Each graph compares the IFN-γ responses of a single patient before and on treatment.
(EPS)

Figure S3 **The gating strategy for detection of virus-specific T cells by dextramer staining.** a) The consecutive gating strategy is shown. Cells were gated for lymphocytes according to forward and side scatter properties, large cells were included to encompass activated and dividing T cells. Dead cells staining positive with a live/dead staining kit were excluded. Cells were further gated on the CD3+ but CD19 negative population, since CD19+ cells can cause non-specific binding. b) CD8+ T cells were stained with a combination of HLA-A2 dextramers loaded with peptides derived from HBV-core, envelope and polymerase (see methods). As a gating control cells were also stained with an HLA-A2 dextramer loaded with an irrelevant peptide.
(EPS)

Figure S4 **The frequency of HBV-dextramer positive T cells is not increased by IL-12 treatment.** Frequency of HBV dextramer+ T cells in 10 day cultures stimulated with HLA-A2 restricted HBV peptides with or without the addition of IL-12.
(EPS)

Figure S5 **IL-12 boosts CD8 T cell responses to epitopes derived from HBV-core, -envelope and -polymerase.** PBMC were stimulated with either, core, envelope or polymerase derived HLA-A2 restricted peptides in the presence or absence of IL-12. CD8 T cells were stained with the corresponding HBV-specific dextramers on day 10 and subsequently restimulated with peptide for 5 hrs. IFN-γ production was detected by intracellular staining and flowcytometric analysis. A representative FACS plot a) and summary data b) for the three patients tested are shown. n.d. signifies no or insufficient dextramer+ cells for analysis could be detected.
(EPS)

Figure S6 **HBV-specific T cells from resolved patients exhibit robust anti-HBV responses that are not significantly boosted by IL-12.** PBMCs from resolved patients were stimulated with HBV-derived peptide in the presence or absence of IL-12 and cultured for 10 days. Peptide-specific CD8 T cells were visualised using HBV-specific dextramers and cells subsequently restimulated with the respective peptides in the presence of brefeldin A. Dextramer+ IFN-γ+ CD8 T cells were quantified by flow cytometry, a) representative FACS plot and b) summary data.
(EPS)

Figure S7 **IL-12 mediated decrease of PD-1 correlates with increased CD8 IFN-γ response.** PBMC were stimulated in short term cultures either with HBV peptide alone or with HBV peptide in combination with IL-12. The fold change of PD-1 MFI on IFN-γ+ CD8 T cells between the two settings is shown, positive values show fold increase, negative values fold decrease, (black bars), and the difference in the % IFN-γ response (%IFN-γ (HBV+IL-12) - % IFN-γ (HBV)) is shown (grey bars). Viral load (log_10_ IU/ml), ALT (IU/L) and patient ID (P. ID).
(EPS)

Table S1 **Table showing patient data for Figure S2.**
(PDF)
