# Supplementary material for: The Third Signal Cytokine IL-12 Rescues the Anti-Viral Function of Exhausted HBV-Specific CD8 T Cells
Source: PLoS Pathog. 2013 Mar 14;9(3):e1003208. doi: 10.1371/journal.ppat.1003208 (PMC3597507; doi:10.1371/journal.ppat.1003208)
Supplement: Table S1 — Table showing patient data for Figure S2. (PDF) [file ppat.1003208.s008.pdf]

Supplementary Table 1

Patient	VL before treatment	VL on treatment	Treatment duration	Patient age	Treatment	HBeAg status
1	4,000,000	63,000	5 month	28	Entecavir	Positive
2	828,700	40	3 month	34	Entecavir	Negative
3	5,501,500	132,500	1 month	67	Entecavir	Negative
4	32,000	332	7 month	57	Lamivudine +Adefovir	Positive
